# The Association Between Screen Time Exposure and Autism Spectrum Disorder-Like Symptoms in Children

**DOI:** 10.7759/cureus.18787

**Published:** 2021-10-14

**Authors:** Nader Alrahili, Najla A Almarshad, Reham Y Alturki, Jamal S Alothaim, Roba Mohsin Altameem, Mohammed A Alghufaili, Abdulmajeed A Alghamdi, Asem A Alageel

**Affiliations:** 1 Department of Psychiatry, Imam Mohammad Ibn Saud Islamic University, Riyadh, SAU; 2 Department of Psychiatry, King Saud University, Riyadh, SAU

**Keywords:** asd-like symptoms, social skills, neurodevelopment, social media, screen time

## Abstract

Research problem

Advances in technology have ensured its inevitable integration in our life. Children, being at a vulnerable age period of development, are spending more time on electronic devices. Some studies reported negative effects on sleep, physical health such as obesity and vision problems, and behavioral changes such as aggressive behavior with exposure to violent media content.

Research significance

We will study the effect of using electronic devices on communication skills in children in Saudi Arabia. Our findings can be used to raise awareness on this matter.

Research objectives

The aim of our study is to examine the association between screen time and social communication skills among children of four years to six years of age in Saudi Arabia.

Research methodology

A cross-sectional study was conducted to investigate the relationship between social skills development and screen time by using a validated Arabic version of the Social Communication Questionnaire (SCQ). The sample in this study consists of 308 children from four to six years of age.

Research results

The results showed that the hours spent using the electronic device were significantly associated with having an SCQ score ≥ 15 (P < 0.05). A high SCQ score was prevalent in 19.7% (n = 31) of children who spent >3 hours using an electronic device compared to 10.2% (n = 5) and 7.84% (n = 8) of children who spent an hour or <2 hours using electronic devices, respectively.

Conclusion

Our study highlighted a significant association between the daily hours spent on devices and having an SCQ score above 15, which suggests a deficit in social skill development and having autism spectrum disorder-like symptoms.

## Introduction

Advances in technology have ensured its inevitable integration in our life. Children, being at a vulnerable age period of development, are spending more time on electronic devices. Some studies have reported negative effects on sleep, physical health such as obesity and vision problems, and behavioral changes such as aggressive behavior with exposure to violent media content [1-3]. However, the benefits of using electronic devices are undeniable. To list a few, electronic devices have facilitated social communication among children in this period of repetitive lockdowns and restricted outdoor activities. In addition, it has revolutionized learning as children have easy access to educational content [3]. Hence, the American Academy of Pediatrics has made age-specific recommendations for media use by children that balance its risks and benefits.

The recommendation on screen time is for caregivers to co-watch good programs with children of 2‒5 years of age for a duration not exceeding one hour a day. Children above 5 years can watch alone while adhering to clear restrictions on screen time and program types that are left to the caregiver to set, avoiding negative effects on the child’s sleep, behavior, or other aspects of health [4]. Similarly, the World Health Organization recommends that children of 4‒6 years of age should not exceed one hour of screen time in a day [5]. The Saudi Ministry of Health makes the same recommendations as the American Academy of Pediatrics guidelines [5].

Healthcare professionals are interested in tracking developmental milestones in children, which can be divided into the following domains: motor skills, social/emotional, language/communication, and cognitive domains. In regard to social development, children of 4‒6 years of age are expected to show interest in other children, play in groups, desire being liked by friends, and understand the concept of cooperation and apology [6]. In contrast, ignoring other children, being excessively shy, and failing to recognize others’ feelings are considered red flags of developmental delay for ages 4, 5, and 6 years, respectively [7].

In regard to language and communication development, children of 4‒6 years of age are expected to be able to tell stories and describe events [6]. If children aged 4, 5, and 6 years fail to answer simple questions, rhyme, and tell clear stories, respectively, they have probably missed a milestone [7]. We postulate that those milestones can be disturbed by increased screen time. Using the Adaptive Social Behavior Inventor, a study found that increased screen time is associated with lower social skills in preschool children [8]. Another study among preschool children found that increased screen time was associated with lower prosocial skills in boys but higher prosocial skills in girls [9].

Moreover, another study conducted among preschoolers in China indicated that the risk of autism spectrum disorder (ASD)-like symptoms increases remarkably in preschoolers whose screen time is >2 hours a day [10]. Another study found that early age of screen exposure increases the incidence of autistic behaviors among preschoolers [11]. A study in Japan showed that the average use of mobile phones was 24 hours per week [12].

A few studies were conducted in Saudi Arabia to identify the most used devices and determine the exposure to these devices. The most used devices are the television, followed by mobile phones and tablets then followed by Computers, In addition children use electronic devices for >4 hours per day [13]. Moreover, there is another study that shows that smartphones are the most common devices used by children (mean usage of 28.5±27 hours per week), followed by tablets (7.5±15 hours per week) and laptops (3±7.4 hours per week). The median duration of use of all the devices together was 35 hours per week [14].

An interesting finding by a study shows that higher-quality screen exposure has been linked to better language skills, whereas higher-quantity screen exposure, introduced early in development, has been linked to lower language skills [15]. There are not enough data on the association between screen time exposure and ASD-like symptoms such as delay in language development, unusual social interactions, odd play patterns, and unusual communication patterns [16].

Our study aimed to examine the association between screen time and social communication skills among children of 4‒6 years of age in Saudi Arabia and identify the factors associated with high SCQ scores.

## Materials and methods

A cross-sectional survey design was conducted to examine the relationship between social skills development and screen time using a validated Arabic version of the Social Communication Questionnaire (SCQ) [17]. The sample in this study consists of 308 children aged 4‒6 years. Data were collected in two steps: first, the researchers shared a form through different social media platforms to collect personal information and contact data of interested participants. Three hundred and eight participants showed interest in participating in this study and the Social Communication Questionnaire (SCQ) was sent to them. The inclusion criteria were children aged 4‒6 years. The exclusion criteria were non-Arabic-speakers and children with developmental disorders or cognitive delay. Also the aim of this study was to study the association between screen time and social communication skills among children of four years to six years of age in Saudi Arabia.

Study design

The Social Communication Questionnaire consists of 40 items. It is a parent-reported scale that can be used to screen for symptoms associated with ASD. We used a validated Arabic version of SCQ with a Cronbach’s alpha coefficient of 0.916, showing high reliability. It also showed high sensitivity and specificity of 0.796 and 0.966, respectively. The 40 items are dichotomous, with only ‘yes’ and ‘no’ as possible answers. Item 1 is only used to assess whether the child can speak with short phrases or sentences, while Items 2 through 40 are used for the actual scoring. Items 2, 9, and 19 through 40 are negatively worded wherein individuals are awarded 1 for response option no and 0 for yes. This reduces the need to reverse the scores. For the other items (i.e., Items 3 to 8 and 10 to 18), a score of 1 is awarded if the answer is yes and 0 otherwise.

Statistical analysis

Statistical analysis was performed using R v 3.6.3. Counts and percentages were used to summarize the categorical variables. The means ± standard deviation or the median/interquartile ranges were used to summarize the distribution of normal and non-normal continuous variables, respectively. The chi-square test was used to investigate the association between categorical variables. Linear regression was used to assess the factors associated with higher total SCQ scores. Hypothesis testing was performed at 5% level of significance.

## Results

Descriptive statistics

The study survey was completed by the caregivers of 308 children (39.6% females and 60.4% males). Approximately half of the respondents were mothers (57.5%) and 17.5% were fathers of the children. The questionnaire was completed by brothers/sisters and aunts in 12.3% and 12.7% of the cases. Respondents aged 4, 5, and 6 years represented 34.7%, 26.3%, and 39% of the study sample, respectively. The majority of the parents for the included children lived in the same house (91.9%). The remaining parents were either separated (6.17%) or widowed (1.95%) (Table 1).

**Table 1:** Descriptive statistics for the study sample

	[ALL]	N
	N=308	
Age:		308
Four years	107 (34.7%)	
Five years	81 (26.3%)	
Six years	120 (39.0%)	
Sex:		308
Female	122 (39.6%)	
Male	186 (60.4%)	
Marital status of caregivers:		308
Parents live in the same house	283 (91.9%)	
Separated	19 (6.17%)	
Widowed	6 (1.95%)	
Relation of caregiver to the child:		308
Aunt	39 (12.7%)	
Brother/Sister	38 (12.3%)	
Father	54 (17.5%)	
Mother	177 (57.5%)	
Education level of caregiver:		308
Bachelor’s degree	195 (63.3%)	
High school	26 (8.44%)	
Middle school	3 (0.97%)	
Post-graduate degree	66 (21.4%)	
Primary school	18 (5.84%)	
The electronic device usually used by the child:		308
Mobile phone	80 (26.0%)	
Other	22 (7.14%)	
Tablet/iPad	124 (40.3%)	
Television	82 (26.6%)	
Does the child have a personal device?		308
No	112 (36.4%)	
Shares with the family	62 (20.1%)	
Yes	134 (43.5%)	
Daily hours spent using the electronic device:		308
Hour or less	49 (15.9%)	
More than three hours	157 (51.0%)	
Two hours	102 (33.1%)	

Regarding the education level of the caregivers, 63.3% of the respondents had completed university education, 21.4% had a postgraduate degree, and 8.44% of the respondents had completed only high school. Tablets and iPads were the main devices used by 40.3% of the children. Mobile phones and televisions were the main devices used by 26% and 26.6% of the children, respectively. Approximately half of the children had a personal device (43.5%); less than one quarter shared devices with the family (20.1%); and 36.4% did not have a personal device. One-half of the respondents (51%) spent more than three hours using the electronic device daily; one-third used it for two hours daily (33.1%); and 15.9% used it for an hour or less daily (Table 1).

**Figure 1:**
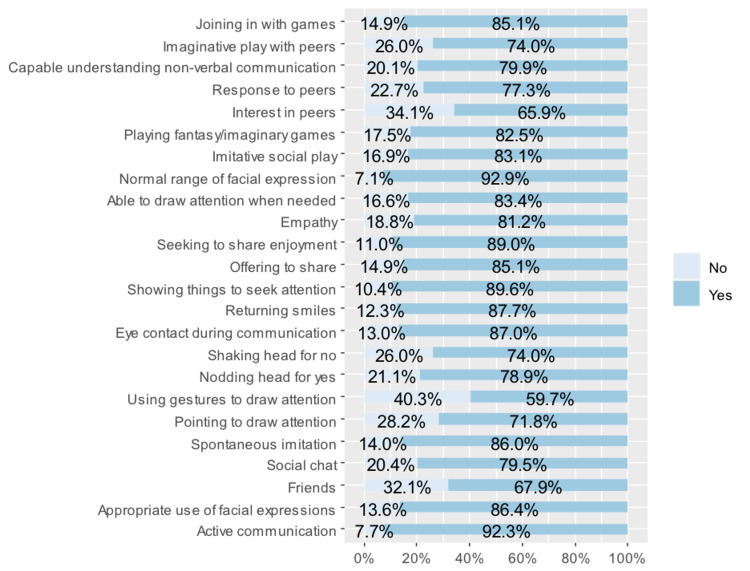
Responses to SCQ items 2, 9, and 19 through 40. SCQ: Social Communication Questionnaire

The results showed that 20.4% of the children had problems with social chat, and 20.1% could not understand non-verbal communication. One-third of the respondents (34.1%) were not interested in peers; a similar number did not have a best friend (32.1%); and 40.3% did not use gestures to draw attention. Only 7.7% of the children had problems with active communication, and only 7.1% did not have a normal range of facial expressions (Figure 1).

**Figure 2:**
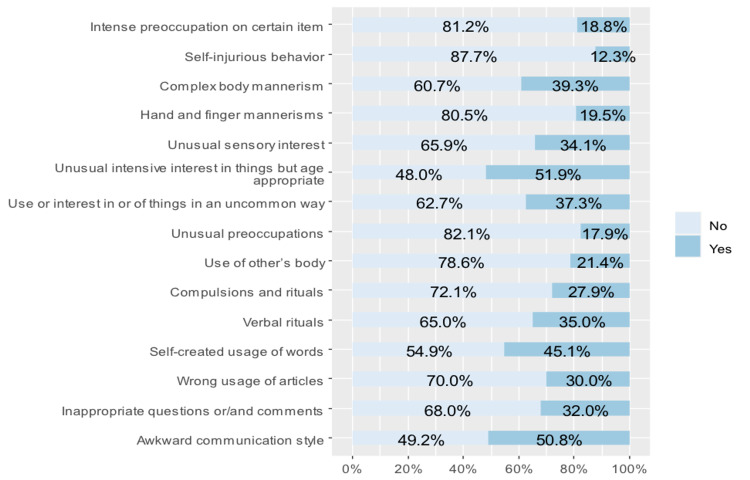
Responses to SCQ items 3 through 8 and 10 through 18. SCQ: Social Communication Questionnaire

The results showed that 50.8% of the children had an awkward communication style; 45.1% used self-created words; and 35% used verbal rituals. Moreover, 51.9% of the children had an unusual intensive interest in things (Figure 2).

**Figure 3:**
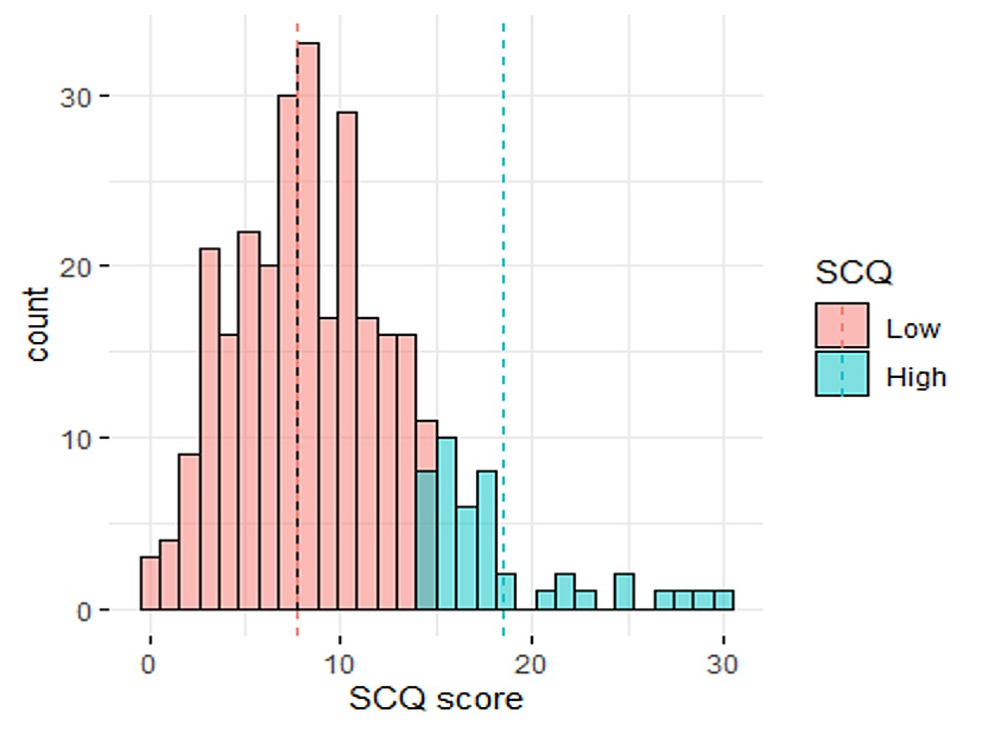
Distribution of SCQ scores. SCQ: Social Communication Questionnaire

The average SCQ score was 9.26 ± 5.19, with minimum and maximum values of 0 and 30, respectively. The distribution was fairly normal. A total of 44 (14.3%) respondents had a high SCQ score, defined as a score ≥15. The chi-square test was used to examine the factors associated with high SCQ scores. These factors included the sex and age of the child, hours spent using electronic devices, ownership of a personal device, and the type of device used (Figure 3).

The results showed that the hours spent using the electronic device were significantly associated with having an SCQ score ≥ 15 (P < 0.05). A high SCQ score was prevalent in 19.7% (n = 31) of children who spent >3 hours using the electronic device compared to 10.2% (n = 5) and 7.84% (n = 8) of children who spent an hour or less and 2 hours using the electronic device, respectively. The marital status and education of the caregiver showed a significant association with a high SCQ score, although the association was significant at the 10% level only. The age and gender of the child were not significantly associated with an SCQ score ≥ 15 (Table 2).

**Table 2:** Factors associated with high SCQ scores Note. Counts and percentages were used to summarize the distribution of categorical variables. Statistical analysis was performed using a chi-square test of independence. SCQ, Social Communication Questionnaire

	Low	High	P
	N=264	N=44	
Age:			0.541
Five years	72 (88.9%)	9 (11.1%)	
Four years	89 (83.2%)	18 (16.8%)	
Six years	103 (85.8%)	17 (14.2%)	
Sex:			0.191
Female	109 (89.3%)	13 (10.7%)	
Male	155 (83.3%)	31 (16.7%)	
Marital status of the caregiver:			0.063
Parents live in the same house	244 (86.2%)	39 (13.8%)	
Separated	17 (89.5%)	2 (10.5%)	
Widowed	3 (50.0%)	3 (50.0%)	
Education level of the caregiver:			0.092
Bachelor’s degree	165 (84.6%)	30 (15.4%)	
High school	21 (80.8%)	5 (19.2%)	
Middle school	2 (66.7%)	1 (33.3%)	
Post-graduate degree	62 (93.9%)	4 (6.06%)	
Primary school	14 (77.8%)	4 (22.2%)	
Electronic device usually used by the child:			0.234
Mobile phone	70 (87.5%)	10 (12.5%)	
Other	20 (90.9%)	2 (9.09%)	
Tablet/iPad	100 (80.6%)	24 (19.4%)	
Television	74 (90.2%)	8 (9.76%)	
Does the child have a personal device?			0.595
No	99 (88.4%)	13 (11.6%)	
Shares with the family	52 (83.9%)	10 (16.1%)	
Yes	113 (84.3%)	21 (15.7%)	
Daily hours spent using the electronic device:			0.019
Hour or less	44 (89.8%)	5 (10.2%)	
Two hours	94 (92.2%)	8 (7.84%)	
More than three hours	126 (80.3%)	31 (19.7%)	

Linear regression (Table 3) was used to examine the factors associated with high SCQ scores (as a continuous variable). So the linear regression analysis showed that gender was significantly associated with the average SCQ score (B = 1.37, P < 0.05), indicating that the average SCQ score is higher by 1.37 points in males than females. Age did not show any significant association with the SCQ score. The time spent using the electronic device was significantly associated with the average SCQ score (B = 1.3, P < 0.05), which indicates that the average SCQ score is higher by 1.3 points in respondents who spent ≥3 hours daily using the electronic device than respondents who used the electronic device for <3 hours daily (Table 3).

**Table 3:** Factors associated with high SCQ scores SCQ: Social Communication Questionnaire

	SCQ score		
Predictors	Estimates	CI	p
(Intercept)	11.44	9.21–13.67	<0.001
Sex: Female	Reference		
Sex: Male	1.37	0.23–2.51	0.019
Caregivers’ Marital status: Married	Reference		
Marital status: Separated	-1.90	-4.22 to 0.42	0.108
Marital status: Widowed	4.64	0.58–8.69	0.025
Electronic device usually used: Mobile	Reference		
Electronic device usually used: Other	-0.01	-2.46 to 2.43	0.992
Electronic device usually used: Tablet/iPad	0.06	-1.38 to 1.49	0.940
Electronic device usually used: Television	-0.25	-1.84 to 1.33	0.756
Child has a personal device: No	Reference		
Child has a personal device: Shares with the family	0.76	-0.87 to 2.38	0.360
Child has a personal device: Yes	0.59	-0.82 to 2.01	0.410
Age: Four years	Reference		
Age: Five years	-0.92	-2.37 to 0.54	0.217
Age: Six years	-0.76	-2.13 to 0.62	0.278
Caregivers’ Education: Bachelor’s degree	Reference		
Education: High school or less	-0.29	-1.94 to 1.35	0.725
Education: Post-graduate degree	-1.27	-2.67 to 0.13	0.075
Daily time spent on the device: <3 h			
Daily time spent on device: ≥3 h	1.30	0.14–2.45	0.028

## Discussion

This study aimed to investigate and provide a new insight into the relationship between prolonged electronic device exposure and autism-like symptoms based on the assumption that excessive use of electronics may lead to ASD-like symptoms. A self-administered questionnaire containing the Social Communication Questionnaire scale (SCQ) was used for preschool children, and other variables added including a question about the hours spent on devices by children.

The results indicate that most of the children used electronic devices for >3 hours daily. Surprisingly, the study highlighted a significant association between the daily hours spent on devices and having an SCQ score above 15, which suggests that children using electronic devices for >3 hours daily may suffer from ASD-like symptoms, indicating the need for further comprehensive clinical evaluation to confirm the diagnosis. Additionally, the results show that the average SCQ score is higher in participants using the electronic devices for >3 hours even if they did not reach the cut-off point of an SCQ score = 15. However, the type of association - causation or coincidence - has not yet been established.

A cohort study of 2152 children was conducted to determine the association between screen time exposure in the first 18 months of life and the development of ASD-like symptoms using the Modified Checklist for Autism in Toddlers (M-CHAT) at two years. This study showed that screen time exposure early in life can be associated with ASD-like symptoms [18]. Interestingly, previous studies concluded that ASD children have a remarkable attraction to screen viewing at a younger age compared to their healthy peers. This is explained by their nature of having less social leisure [19] and preference for computing activity over social network browsing because it is very demanding for them to be socially engaged [20].

Moreover, previous research has proven that the severity of ASD symptoms is proportionate to screen time, clearly recognized in atypical sensory responsiveness and greater susceptibility to developmental delay, specifically in the language domain [21]. Simultaneously, the reverse casualty must be considered. Our data failed to show a significant relationship between an SCQ score above 15 and the marital status and education of the caregiver or the age and gender of the child. Contrary to our expectations, although the majority of the participants’ caregivers was well educated and expected to be aware of the risk of overexposure, most of the children had personal devices, with the tablet being the most common. Therefore, it is crucial to emphasize the risk and benefits of these electronic gadgets.

In line with our hypothesis, a large-scale study conducted in Korea on toddler children to determine the effect of screen time on language development through multiple interviews and questionnaires found that language delay is proportional to screen time, with a 2.7 times greater risk for those who spend 2 hours of watching television [22]. The reliability of this data is impacted by several factors.

Limitations

First, data was collected during the COVID-19 pandemic when all schools and kindergartens had shifted to virtual classes and activities. Second, this study was subject to reporter bias as it relies on caregiver recall. Third, our study was limited to caregiver who uses social media.

## Conclusions

This study has shown that there is a significant association between daily hours spent on devices especially three hours and more and having an SCQ score above 15, which may suggest a possible ASD-like symptoms. However, the type of association has not yet been established. Our findings can be used to raise awareness on this matter and help set guidelines on the use of media for children. Future studies are required to assess association.
